# Advanced setup for safe breath sampling and patient monitoring under highly infectious conditions in the clinical environment

**DOI:** 10.1038/s41598-022-22581-7

**Published:** 2022-10-26

**Authors:** Pritam Sukul, Phillip Trefz, Jochen K. Schubert, Wolfram Miekisch

**Affiliations:** Rostock Medical Breath Research Analytics and Technologies (ROMBAT), Department of Anaesthesiology and Intensive Care, University Medicine Rostock, Schillingallee 35, 18057 Rostock, Germany

**Keywords:** Biomarkers, Translational research, Analytical chemistry, Infectious-disease diagnostics, Mass spectrometry, Metabolomics, Risk factors

## Abstract

Being the proximal matrix, breath offers immediate metabolic outlook of respiratory infections. However, high viral load in exhalations imposes higher transmission risk that needs improved methods for safe and repeatable analysis. Here, we have advanced the state-of-the-art methods for real-time and offline mass-spectrometry based analysis of exhaled volatile organic compounds (VOCs) under SARS-CoV-2 and/or similar respiratory conditions. To reduce infection risk, the general experimental setups for direct and offline breath sampling are modified. Certain mainstream and side-stream viral filters are examined for direct and lab-based applications. Confounders/contributions from filters and optimum operational conditions are assessed. We observed immediate effects of infection safety mandates on breath biomarker profiles. Main-stream filters induced physiological and analytical effects. Side-stream filters caused only systematic analytical effects. Observed substance specific effects partly depended on compound’s origin and properties, sampling flow and respiratory rate. For offline samples, storage time, -conditions and -temperature were crucial. Our methods provided repeatable conditions for point-of-care and lab-based breath analysis with low risk of disease transmission. Besides breath VOCs profiling in spontaneously breathing subjects at the screening scenario of COVID-19/similar test centres, our methods and protocols are applicable for moderately/severely ill (even mechanically-ventilated) and highly contagious patients at the intensive care.

## Introduction

Breathomics offers non-invasive phenotyping and monitoring of systemic physio-metabolic^1–4^, pathological^5–8^ and therapeutic^9,10^ effects. Being the proximal matrix, breath holds instant/local metabolic information on respiratory infections and/or co-infections^11,12^. Moreover, bronco-pulmonary exchange of blood-borne volatile metabolites projects further non-invasive insight into deeper pathobiological cascades at the organ or even at the cellular levels^13,14^.

Despite such attractiveness, breath sampling in highly contagious respiratory infections e.g. common cold, influenzae, bronchitis, pneumonia and especially COVID-19 impose critical challenges to the present state-of-the-art for sampling and analysis. Such infections being contagious via breath^15,16^, shouts for sampling and analytical methodology with reduced-risk of pathogen transmission.

We have witnessed the ubiquitous hurdles faced by the scientists and/or clinicians while dealing with SARS-CoV-2 infected subjects^17,18^. Mandatory safety and precaution measures have often overruled/compromised sampling and analytical requirements/standards, which have affected the quality, reliability and reproducibility of obtained data^19,20^. Meanwhile, researchers tried various analytical methods to detect COVID-19 infection via breath. Such approaches implemented sensor array based single-point analysis of signal patterns or features^21,22^ from COVID-19 cases, analysis of SARS-CoV-2 load in exhaled breath condensate^23^ as well as canine driven detection of odours from infected patient masks^24^ etc. Nonetheless, the actual identification and quantification of exhaled end-tidal VOCs concentrations is challenging. Although a recent study has well addressed online mass-spectrometry based analysis of breath samples, collected in 1L Tedlar bags, the scope and potential of direct sampling (i.e. without collection and storage) and breath-resolved analysis remained unaddressed and unexplored. Another study demonstrated Fourier-transform infrared (FTIR) spectroscopy and artificial intelligence-based assessment of breath VOCs collected in Tedlar bags^25^. Though these punctual samples were cooled at – 20 °C before analysis, the obvious condensation, clustering and loss of volatile substances were not addressed or considered. Similarly, offline sampling in Tedlar bags, Tenex tubes and analysis protocols via thermodesorption based gas-chromatography mass-spectrometry are proposed^26^. Nevertheless, authors could not introduce viral filters within their workflow due to the complex artefacts induced by such filters and those errors remained untraceable via offline methods. An optimized methodology and/or protocol for reliable and repeatable real-time and offline breathomics under COVID-19 condition as well as additional mandatory safety measures will induce analytical confounders and will increase systematic errors. For instance, we have recently demonstrated immediate and progressive effects of medical face-masks (surgical and FFP2) on exhaled VOCs compositions in healthy adults aged between 20 and 80 years^27^. Therefore, measurements within mask space cannot be attributed for reproducible sampling.

In order to resolve those issues, we need easy to use point-of-care applicable sampling methods with minimal-risk of infection/transmission while screening large population for infections such as COVID-19 and while measuring SARS-CoV-2 positive patients with moderate symptoms/SARS-onset or under mechanical ventilation. In order to guarantee the valid scientific outcomes, reliable protocols and methods for sampling and analysis of breath via real-time and lab-based techniques have to be applied.

Thus, general requirements for the experimental setup, customization/modifications of sampling devices and analytical tools as well as use of personal protective equipment (PPE) are to be well-defined for both online and offline analysis. In line with the urgent needs of the breath- and respiratory research community, we are addressing safe, reliable and repeatable breath sampling procedures and operational conditions in patients infected with SARS-CoV-2 and/or other respiratory pathogens. The following issues will be addressed explicitly:

- Suitability of viral filters for point-of-care (PoC) and lab-based breath sampling and analysis.
- Comparisons of confounding filter effects (physiologically and analytically).
- Optimizations of PoC breath sampling methods for the SARS-CoV-2 test-centre and intensive care.
- Recommendations for online and offline breathomics under infectious / COVID-19 safety condition.

## Results

**Figure 1:**
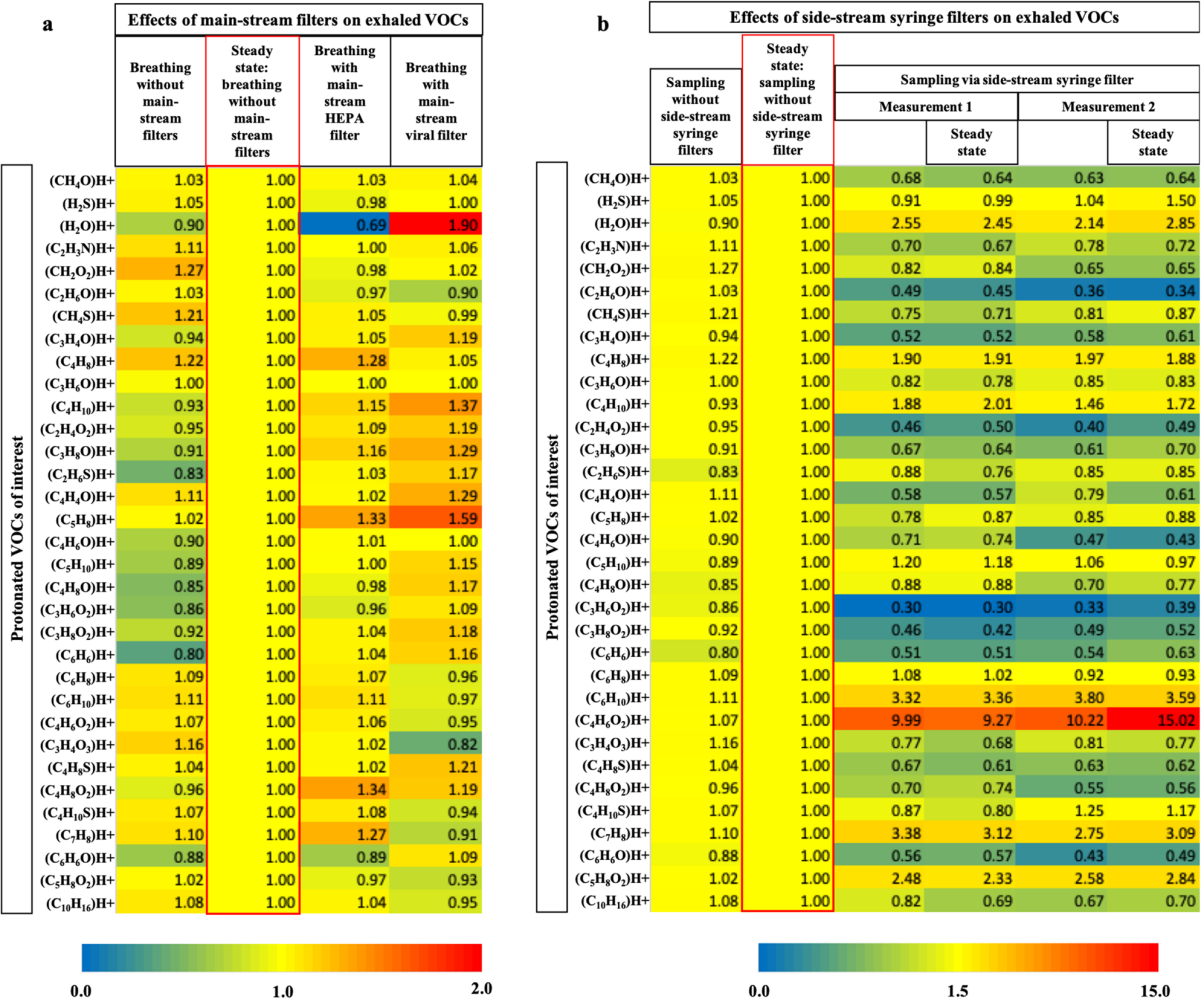
Relative differences in exhaled VOCs concentrations (mean over two min) without and with the mainstream (**a**) and side-stream (**b**) viral filters. All Y-axis represent protonated VOCs of interest. All X-axis represent experimental conditions. VOCs data were normalised onto the corresponding values from sampling at the steady state of breathing without filters that are placed within red-coloured margins. The steady states are the actual comparison points for physiological and/or analytical effects induced by the filters. Changes in colour represents relative differences in concentrations. Red and blue colour represents relatively high and low values, respectively. (**a**) Mainstream filters depict both physiological and analytical effects. (**b**) Side-stream filters depict only analytical effects and repeated measurements (1 and 2) are presented to demonstrate repeatability.

Figure 1 represents immediate effects of viral filters on exhaled VOCs profiles. Figure 1a represents heatmaps of relative differences in exhaled VOCs concentrations (mean over two min) between real-time sampling without and with two different mainstream filters and Fig. 1b represents the same between real-time sampling without and via side-stream syringe-filter. Repeated measurements demonstrate repeatability. Statistical significances of observed differences in Fig. 1 are presented in Supplementary Fig. S1 online. Contribution and loss of VOCs via mainstream and side-stream filters under different sampling flows are presented as the heatmaps in Supplementary Fig. S2 online.

**Figure 2:**
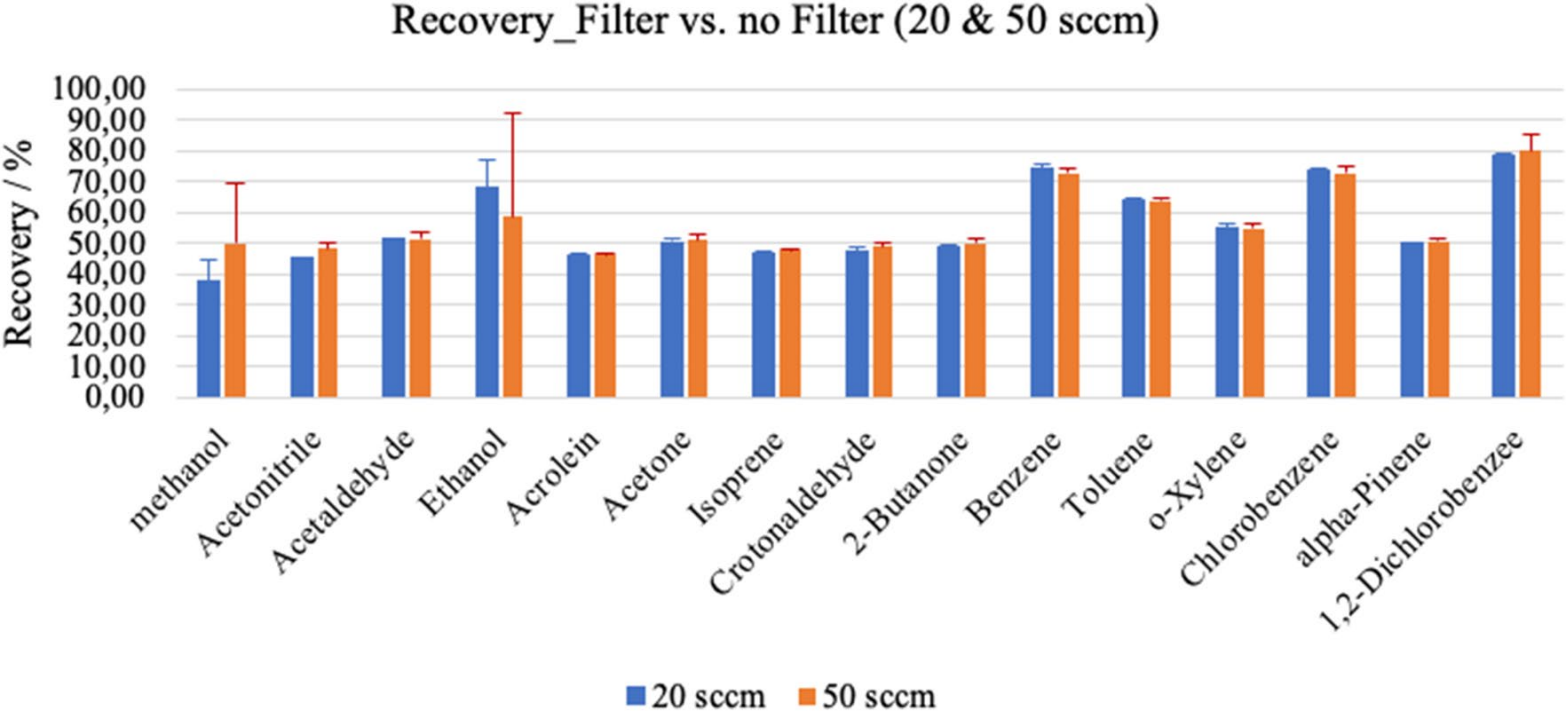
Recovery rates of VOCs concentrations with side-stream syringe filters at two different sampling flow rates. VOCs intensities obtained through measurements without filters represent 100%, VOCs intensities obtained through measurements with syringe filter are presented in % in relation to measurements without filter. VOCs concentration was approximately 100 ppbV. Blue bars represent measurements with a sampling flow rate of 20 sccm (i.e. mL/min), orange bars represent measurements with a sampling flow rate of 50 sccm.

Figure 2 represents recovery rates of VOCs with side-stream syringe-filter in comparison to measurements without filter under sampling flow rate of 20 sccm (i.e. mL/min) and 50 sccm. Any considerable difference in flow dependency of VOCs concentrations was not observed between inlet flow rate of 50 sccm and 65 sccm (Supplementary Fig. S3 online).

VOCs intensities and relative standard deviations (RSDs) obtained through repeated measurements under different concentrations (2–100 ppbV) with side-stream syringe filters at those two sampling flow rates are presented in Table 1.

**Table 1:** VOCs intensities and relative standard deviations (RSDs) from repeated measurements with side-stream syringe filters at two different sampling flow rates.

			Peak areas/counts (cps)						Relative standard deviations/%					
	Concentrations (ppbV)		2.0	5.0	10.0	25.0	50.0	100.0	2.0	5.0	10.0	25.0	50.0	100.0
	VOCs	Mass												
VOCs Mix_Humid_filter_20sccm	Methanol	33.03	155.5	170.6	211.4	303.6	448.8	499.5	5.0	22.4	41.8	56.2	51.9	18.8
	Acetonitrile	42.04	90.4	136.3	209.0	430.1	809.4	1502.5	3.7	6.3	10.0	7.7	1.0	0.3
	Acetaldehyde	45.03	214.2	247.5	321.4	533.5	876.7	1552.1	1.0	1.3	3.1	0.1	0.2	0.3
	Ethanol	47.05	299.1	286.1	293.9	290.0	323.2	313.9	4.2	3.6	10.3	16.8	18.1	13.1
	Acrolein	57.03	55.0	104.8	188.2	432.9	862.9	1690.6	2.5	0.5	2.0	1.0	0.3	1.3
	Acetone	59.05	185.7	236.6	331.6	615.8	1087.0	1978.9	2.5	2.9	3.4	0.5	2.2	1.6
	Isoprene	69.07	32.6	54.8	91.4	197.2	376.7	748.5	1.1	2.2	2.5	1.1	1.0	1.8
	Crotonaldehyde	71.05	58.7	120.5	212.8	520.6	1034.3	2064.6	5.7	3.8	10.2	5.2	0.1	2.6
	2-Butanone	73.06	74.7	126.5	212.9	475.7	920.8	1819.4	2.9	2.7	2.0	0.3	2.4	1.3
	Benzene	79.05	29.2	65.0	121.5	314.1	633.3	1292.4	0.0	1.4	3.2	1.6	0.9	1.1
	Toluene	93.07	49.4	86.7	148.5	338.1	672.9	1399.5	4.0	0.2	0.6	1.0	0.3	0.6
	o-Xylene	107.09	31.6	65.0	131.1	316.8	667.1	1432.7	4.2	0.3	0.6	1.3	0.7	2.2
	Chlorobenzene	113.02	37.6	66.1	116.8	291.3	568.5	1177.6	5.7	6.1	3.1	0.4	0.3	1.4
	alpha-Pinene	137.13	13.6	25.4	47.3	118.2	255.9	553.1	5.0	2.9	2.4	0.6	1.7	0.8
	Dichlorobenzene	148.00	2.5	5.0	7.5	18.7	33.9	62.9	4.1	1.4	16.4	13.2	7.2	1.2

	Concentrations (ppbV)		2.0	5.0	10.0	25.0	50.0	100.0	2.0	5.0	10.0	25.0	50.0	100.0
	VOCs	Mass												
VOCs Mix_Humid_filter_50sccm	Methanol	33.03	142.0	171.0	224.5	333.0	528.9	663.9	10.7	32.8	56.7	66.4	66.9	39.0
	Acetonitrile	42.04	70.5	123.5	204.2	439.1	826.3	1590.8	3.1	12.6	18.8	13.3	5.4	4.9
	Acetaldehyde	45.03	174.9	217.7	287.6	504.4	841.0	1546.1	0.4	6.0	1.9	8.3	3.2	3.6
	Ethanol	47.05	117.6	128.5	143.0	171.4	217.8	270.6	15.9	26.6	47.1	55.2	63.2	56.2
	Acrolein	57.03	61.1	107.3	197.7	451.3	878.8	1715.5	4.7	0.6	2.1	1.4	1.3	0.6
	Acetone	59.05	178.3	237.8	330.3	607.5	1069.8	1995.2	1.0	8.4	2.1	4.7	2.5	3.5
	Isoprene	69.07	33.9	53.0	92.0	196.6	379.5	765.4	3.2	1.8	1.2	4.7	1.6	1.4
	Crotonaldehyde	71.05	64.3	127.6	228.6	544.1	1070.0	2138.0	0.6	2.0	12.5	8.9	4.4	2.9
	2-Butanone	73.06	56.2	109.1	197.9	466.1	913.8	1850.7	14.9	2.5	7.9	3.8	0.5	3.1
	Benzene	79.05	30.0	62.4	119.1	309.6	616.3	1261.3	0.9	1.7	2.5	5.2	1.9	1.7
	Toluene	93.07	41.3	76.5	144.2	331.5	660.9	1391.7	3.5	4.9	2.9	2.6	1.6	1.0
	o-Xylene	107.09	29.1	66.7	129.4	321.1	667.0	1428.2	1.9	4.1	2.4	6.0	0.3	2.1
	Chlorobenzene	113.02	36.3	63.4	122.5	279.7	568.6	1168.8	6.8	6.1	0.7	2.0	0.9	3.1
	alpha-Pinene	137.13	12.4	26.2	47.0	123.6	259.7	561.8	3.5	6.1	1.6	4.5	1.9	0.9
	Dichlorobenzene	148.00	2.5	4.5	8.2	18.8	33.0	64.1	29.0	1.1	15.9	10.3	7.7	6.6

VOCs intensities (cps) along with corresponding RSDs (%) obtained through repeated measurements under different concentrations (2–100 ppbV) with syringe filters are presented. Data represent measurements with sampling flow rates of 20 sccm and 50 sccm.

Comparisons between absolute abundances of exhaled VOCs measured repeatedly by our customised viral filter attachments are presented in Supplementary Table S1 online. Mean changes (and SDs) in most of the endogenous VOCs remained < 10% and overall, no significant differences were observed within the percentage of differences between repeated measures in individuals.

The effects of continuous breath-resolved measurement time on the stability of the syringe-filters are presented in Supplementary Fig. S4 online. Distinct separations between inspiratory and expiratory breath phases starts to dilute after the 5th minute.

**Figure 3:**
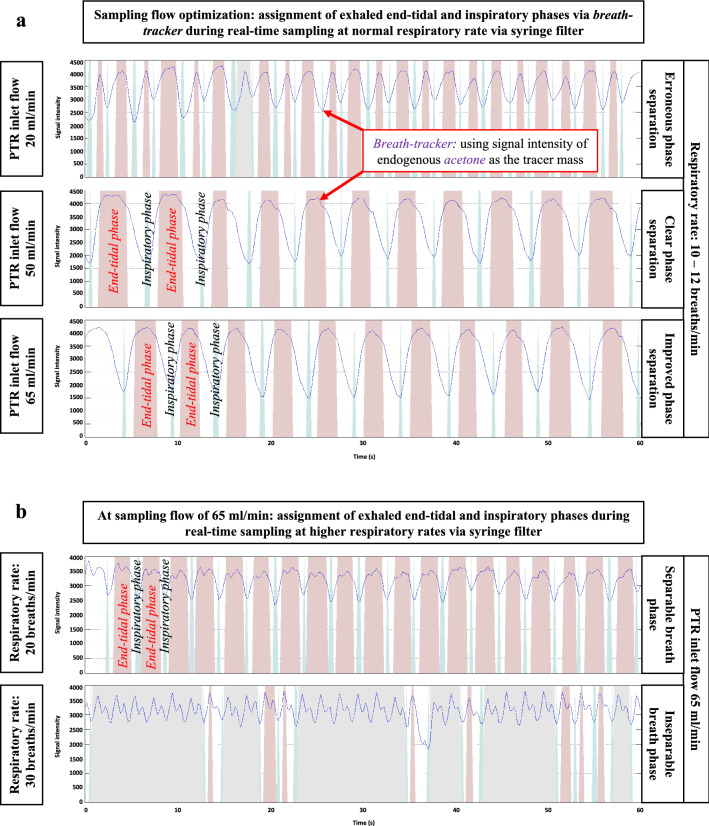
Effects of PTR sampling flow on breath-resolved assignment of expiratory and inspiratory phases via syringe-filters under normal (**a**) and higher (**b**) respiratory rates: Here, *breath tracker* plots are presented under various sampling conditions. In all breath-tracker plots, Y-axis represent signal intensity of acetone (an endogenous and blood-borne VOCs) and all X-axis represent time in s. Red colour represents exhaled alveolar/end-tidal phase and blue colour represents inspiratory (room-air) phase. (**a**) Sampling flows of 20, 50 and 65 mL/min were applied for sampling under respiratory rate of 10–12 breaths/min. (**b**) Sampling flow of 65 mL/min was applied for sampling high respiratory rates of 20–30 breaths/min.

Figure 3 represents effects of PTR sampling flow on breath-resolved assignment of expiratory and inspiratory phases. Figure 3a represents side-stream sampling at different flows via syringe-filters under normal respiratory rate and Fig. 3b represents side-stream sampling at high flow rate via syringe-filters under higher respiratory rate.

**Figure 4:**
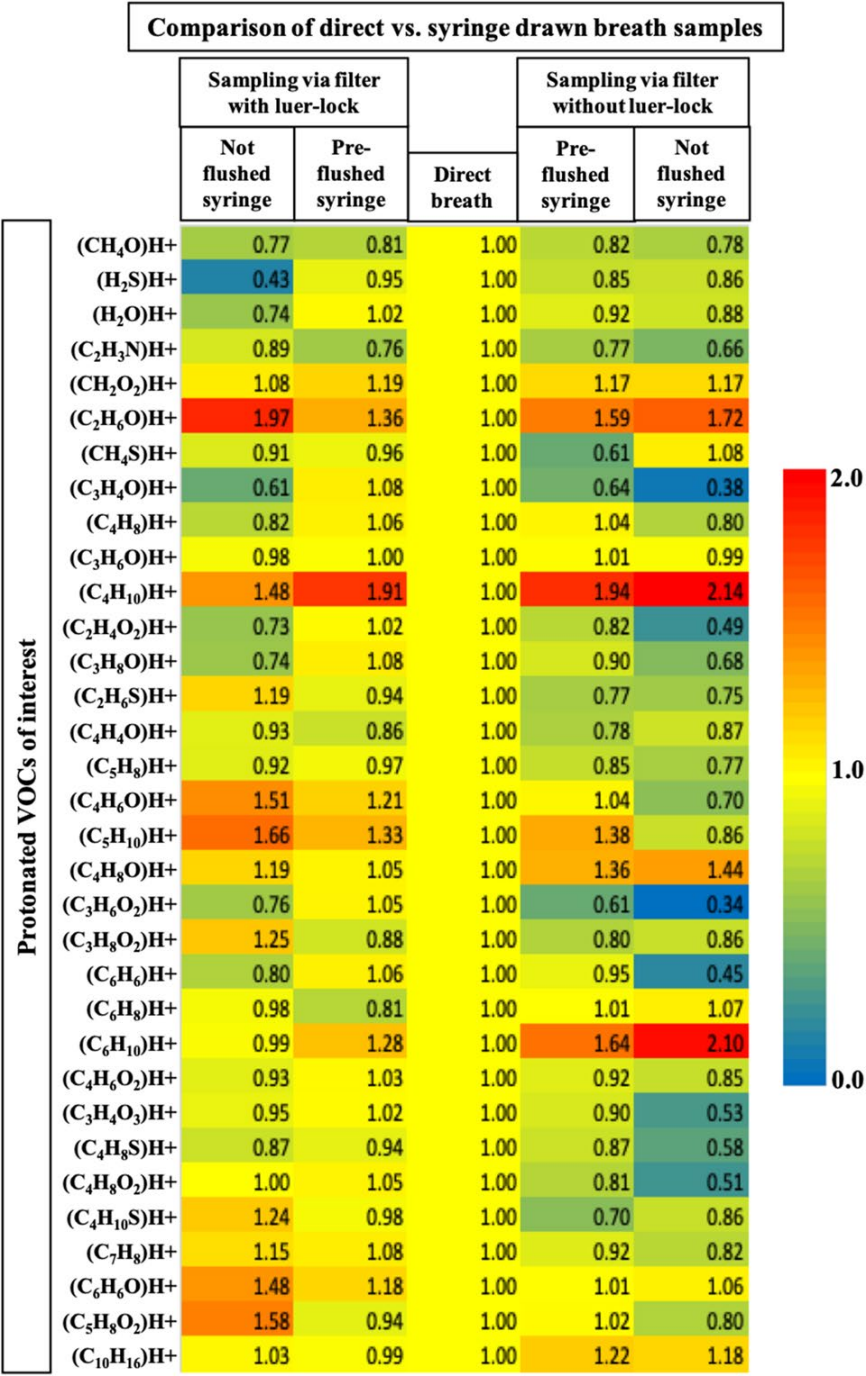
Effects of viral filters on side-stream manual sampling with different connection types with and without pre-purging: syringe drawn breath was analysed via PTR-ToF–MS and compared to direct PTR-ToF analysis. Y-axis represents protonated VOCs of interest. X-axis represents experimental conditions. VOCs data from syringe samples were normalised onto the corresponding values without filter. Syringe filters with and without luer-lock were compared. Effect of flushing and not flushing syringes prior to sampling are observed. Changes in colour represents relative changes in concentrations. Red and blue colour represents relatively high and low values, respectively.

Figure 4 represents heatmaps of relative differences in VOCs concentrations between the original breath (i.e. direct breath) and 50 mL of the same breath i.e. sampled in glass-syringe via different types of syringe filters. Analytical effects of not flushing vs. pre-flushing (in and out) with one exhalation prior to actual sample are presented. Statistical significances of observed differences are presented in Supplementary Fig. S1 online.

**Figure 5:**
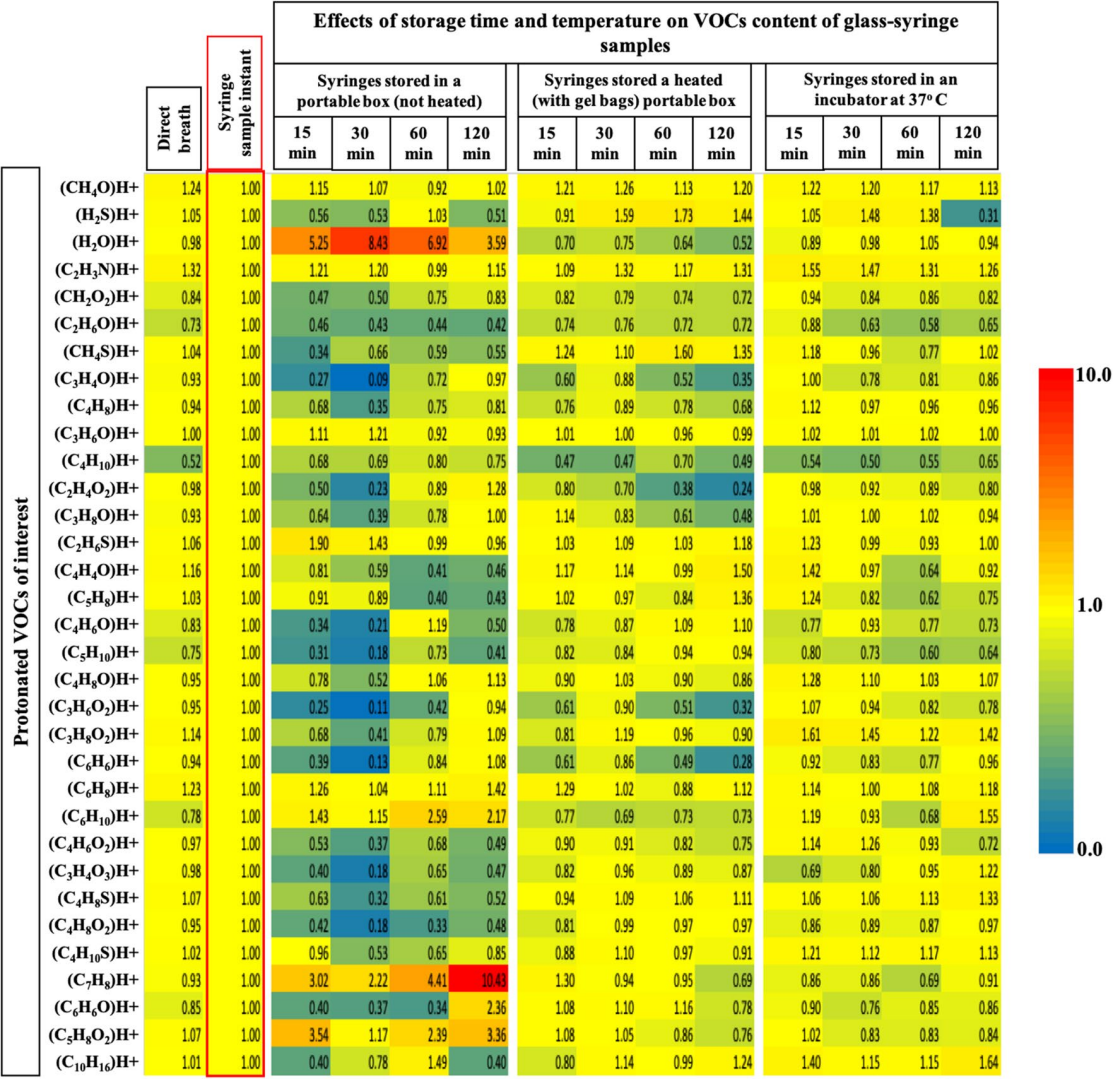
Effects of storage time and temperature of glass-syringe samples onto the VOCs concentrations: Y-axis represents protonated VOCs of interest. X-axis represents experimental conditions. VOCs data (from repeated measurements) were normalised onto the corresponding values from the syringe sample i.e. analysed instantly (without storing). Values from direct breath are depicting syringe-filter driven analytical variations. Effects of storage time of 15, 30, 60 and 120 min on VOCs content are presented along with the effects of storage (for transport) temperature (not heated and heated at 37 °C).

Figure 5 represents heatmaps of relative differences in VOCs concentrations of glass-syringe samples stored up to 120 min under heated (at 37 °C) or not heated conditions. Syringe-filter driven analytical variations are presented in comparison to corresponding values directly from the breath (from which the syringe was actually sampled). Statistical significances of observed differences are presented in Supplementary Fig. S1 online.

## Discussion

At the precedent International Breath Summit 2021 (organised by the *International Association of Breath Research—IABR)*, and at the ERS Congress 2021 (organised by the *European Respiratory Society—ERS)*, rising concerns were observed on the available state-of-the-art methods and protocols for real-time and offline breath sampling and analysis in COVID-19 patients with minimal risk of viral transmission. Estimation of physiological and/or analytical confounders related to sampling and analysis are indispensable prerequisites for valid clinical interpretations of measured breath biomarkers^28,29^. Such factors may easily override actual pathobiological effects if not considered. As real-time mass-spectrometry offers direct analysis of breath, which is convenient, fast and suitable for screening large population, a series of parameters were optimized and explored for this purpose. Nevertheless, online analysis is difficult to implement at the bedside e.g. in severely ill or mechanically ventilated patients. Therefore, methods of offline sampling and analysis were examined, improved and optimised for offline analytical techniques such as microextractions coupled with GC–MS or GCxGC-ToF–MS, which is a gold-standard for identifications and quantifications of known/unknown trace VOCs. We observed both physiological and analytical effects of the unavoidable COVID-19 safety measures on exhaled breath biomarkers resulting in immediate changes in VOCs contractions. Such effects were substance specific and depended on the origin and physio-chemical properties of VOCs. Immediate physiological effects were observed in case of main-stream viral filters due to implied upper-airway resistance against breathing. In case of side-stream syringe-filters, only analytical effects were observed and those effects were systematic. Effects partially depended on the sampling flow and respiratory rate. For syringe sampling factors such as storage time, -conditions and -temperature played important role. Contributions or losses of VOCs via the filters, filter’s performances, stability and variability in repeated measurements were examined for online and offline analysis.

In order to prevent any infectious contamination to/from the room air, application of a main-stream viral filter at the rare end of the breathing mouth-piece is mandatory. Previously we observed immediate effects of increased upper-airway resistances on exhaled VOCs profiles of healthy subjects^30^. In line with those observations, both mainstream filters induced effects such as altered alveolar plateau at expiration, shift of pulmonary diffusion gradients at inspiration, collateral alveolar ventilation and respiratory mechanics onto VOCs profiles. Therefore, substances with higher vapour pressure depicted increased alveolar elimination under mainstream filters. While compared to the steady state of breathing^2^ without any filter, the physiological effects induced by HEPA filters were less pronounced than that of the other mainstream viral filter. These minimal effects from HEPA filter should be considered as unavoidable systemic confounders for breath analysis under the pandemic situation. Alveolar elimination of endogenous substances with relatively higher vapour pressure (e.g. C_5_H_8_ and C_2_H_6_S) were increased due to inspiratory resistance driven negative intrathoracic pressure. HEPA filters absorbed humidity and water-soluble compounds like H_2_S, CH_2_O_2_, C_3_H_6_O. It also accumulated compounds such as C_3_H_8_O, C_6_H_6_, and C_7_H_8_. While compared to room air measurements and breathing without filter, the origins of increased C_4_H_10_ and C_4_H_8_O_2_ concentrations must be assigned to the HEPA filters. A small number of other VOCs are also contributed or absorbed by the HEPA filters and those compounds should be evaluated carefully during clinical interpretation of results.

Side-stream syringe-filters were used to prevent viral transmission to the sampling and/or analytical equipment. These filters introduced various analytical effects and repeated measurements of matrix-adapted humid standard VOCs mixtures depicted substance specific recovery rate along with effects of sampling flow. For instance, increased RSDs in aliphatic alcohols can be attributed to condensation effects from the humid layer (that accumulates over time) of these filters onto such polar compounds. Those effects were similar, systematic and reproducible during the actual breath measurements via side-stream filters, while compared to corresponding values of VOCs at the steady state of breathing without filters. Noteworthy that the differences in recovery rate of actual breath measurements are to be attributed to the physiological effects induced in parallel by the mandatory mainstream HEPA filter. Accumulation of humidity and substances such as C_4_H_10_, C_6_H_10_, C_4_H_6_O_2_, C_7_H_8_ and C_5_H_8_O_2_ were observed in breath profiles. While compared to ambient air and breathing without filter, C_6_H_10_ and C_4_H_6_O_2_ are clearly perceived to originate from the side-stream filters. A large number of other VOCs are also contributed or absorbed by the syringe-filters, which may mislead clinical interpretation of these substances as biomarkers—if not considered meticulously.

As our standard practice for real-time measurements in spontaneously breathing humans, we use 20 mL/min of side-stream sampling flow for breath-resolved assignment of VOCs^7,31,32^. While introducing the side-stream syringe-filter, only increased sampling flows could offer good separation between inspiratory and expiratory phases under normal breathing. As, phase separation starts to dilute with the increase in respiratory rate, a high sampling flow of 65–100 sccm is recommended to attain breath-resolved measurements of VOCs without compromising the analytical sensitivity.

Inspiratory and expiratory phases start to dilute as the side-stream filter gets humid over time. Therefore, a distinct assignment of alveolar VOCs concentrations is only feasible for 5 min and a new filter should be used at this point.

By applying mainstream HEPA and side-stream syringe filters, VOCs concentrations were affected diversely (physiologically and analytically) in real-time. As these effects were systematic and did not change significantly under repeated measurements, repeatable online sampling and robust analysis is feasible under the unavoidable safety measures against infection transmission. Using higher sampling flow offered better assignment of end-tidal and inspiratory phases at respiratory rate < 20 breaths/min.

In case of manual side-stream alveolar sampling (CO_2_ controlled)^33^ in 50 mL glass syringes, the filters with male Luer-lock adapter turned out to be best option. Flushing of the glass-syringes via 1–2 exhalations before drawing the actual sample, reduced the confounding effects from the filter dead space. Once these 50 mL samples were compared directly to the breaths from which they were actually sampled, the differences in most of the VOCs remained < 10%. As 50 mL syringe was drawn manually within 1 s at the alveolar phase (by following the capnograph) of an exhalation, most of the confounding analytical effects were less pronounced in such punctual sampling. This allows near-breath quantification of the VOCs concentrations whereas, real-time measurements offer immediate and continuous assessment of relative changes due to physiological and/or analytical effects. It is also possible to conduct parallel measurements by using our ‘combined sampling attachments’, which will allow cross-validation of methods.

Optimal conditions for storage and transport of syringe samples from clinical setup to mass-spec laboratory are crucial^34,35^. While storing the samples in Styrofoam box, heating of the box has shown better stability of samples over time. If not heated, syringes start to have condensation (we have observed vapour accumulation on the inner wall of the syringes) and therefore certain VOCs start to accumulate or dilute (/cluster with the vapour)^36^. Heating of glass syringes with gel-bags (heated at 37 °C) have shown good stability up to one hour. Keeping the samples in an incubator (at 37 °C) depicted best quality of samples till 2 h. The differences between results from the gel-bags and an incubator were mainly due to the distribution of heat. Where possible, we recommend to keep the sampled syringes in an incubator for 15 min prior to analysis.

Based on the above-discussed facts and experiences^37^, we hereby recommend the following setups for safe real-time and offline breath VOCs measurements under highly infectious conditions (Schematic overview is presented in Supplementary Fig. S5 online). In both cases, investigator/operator should wear the recommended personal protective equipment (PPE) at all time. In order to avoid cross infection (between participants), subjects should use COVID-19 protective medical face-masks while waiting in the test queue (by maintaining a minimum distance of 2 M from each other) and should disinfect their hands at the entrance of the SARS-CoV-2 test center. After entering the sampling area healthy subjects or patient (asymptomatic or mildly symptomatic) should sit on a disinfected chair, remove his/her face masks and orally breathe in and out through the sterile mouthpiece of our customised sampling attachment. Participants should not touch any instrumental parts by hand except encircling the breathing-end of the sterile mouthpiece via his/her lips. Volunteers should follow our standard sampling manoeuvre^2^. After sampling subjects should immediately put on their face-masks and disinfect their hands again at the exit of the test center. Used sampling attachments (mouthpieces and filters) and all disposable materials must be replaced after single use. The sampling area must be disinfected and adequately ventilated before and after each participation.

For real-time measurements, the area of analytical instrument and its operator should be separated from the patient/subject entry and sitting area via a transparent wall. Only the PTR-transfer-line should enter towards patient’s side via a custom-made entry hole of the transparent wall. The transfer-line should be enclosed by a sterile and disposable cover that must be disposed after each use.

For offline measurements, a transparent Teflon-sheet made protective separation should be used to separate the mouthpiece parts at test subject’s end from the sampling parts at the operator’s side. Syringes should be flushed-in and -out with two exhaled breaths before taking the actual alveolar sample to avoid dilution effects. Immediately after sampling, syringes should be closed via an adapter-system and then detached from the syringe-filter for storage/transport. Three or more syringes shall be sampled from each subject. Corresponding room air should be sampled (via syringe-filter) separately for comparison of inspiratory VOCs concentrations.

Our methods provide repeatable conditions for point-of-care and lab-based breath sampling and analysis with low risk of disease transmission under COVID-19 and similar infectious conditions. Besides breath VOCs profiling in spontaneously breathing subjects (healthy, asymptomatic positive and mildly-symptomatic patients) under the screening scenario of a COVID-19 test centre^37^, the above methods and protocols are applicable for monitoring moderately-symptomatic (with SARS onset) and/or severely ill mechanically-ventilated patients at the COVID-19 intensive care unit (ICU).

## Methods

All methodological and analytical optimizations were carried out in accordance with the Good Laboratory Practice (GLP) guidelines and all clinical experiments were conducted according to the amended Declaration of Helsinki (DoH) guidelines. As mandatory prerequisites, ethical approvals (Approval no: *A2020-0085* and *A2021-0012*) from the Institutional Ethics Committee (IEC) of the University Medicine Rostock (Rostock, Germany) and signed informed consent from all subjects were obtained.

In compliance with the unavoidable infection safety mandates, we have advanced our state-of-the-art experimental setups and methods. In order to reduce viral transmission risk, we have tested both mainstream and side-stream viral filters with pore size of 0.2 μm. Mainstream filters were to avoid viral contamination of the clinical environment, side-stream filters were used to protect sampling and analytical equipment. Feasibility and confounding effects of applying such filters (along with our optimised methods, setup and analytical parameters) in clinical studies were examined on 10 healthy adults (5 male and 5 females, aged between 18 and 70 years).

**Figure 6:**
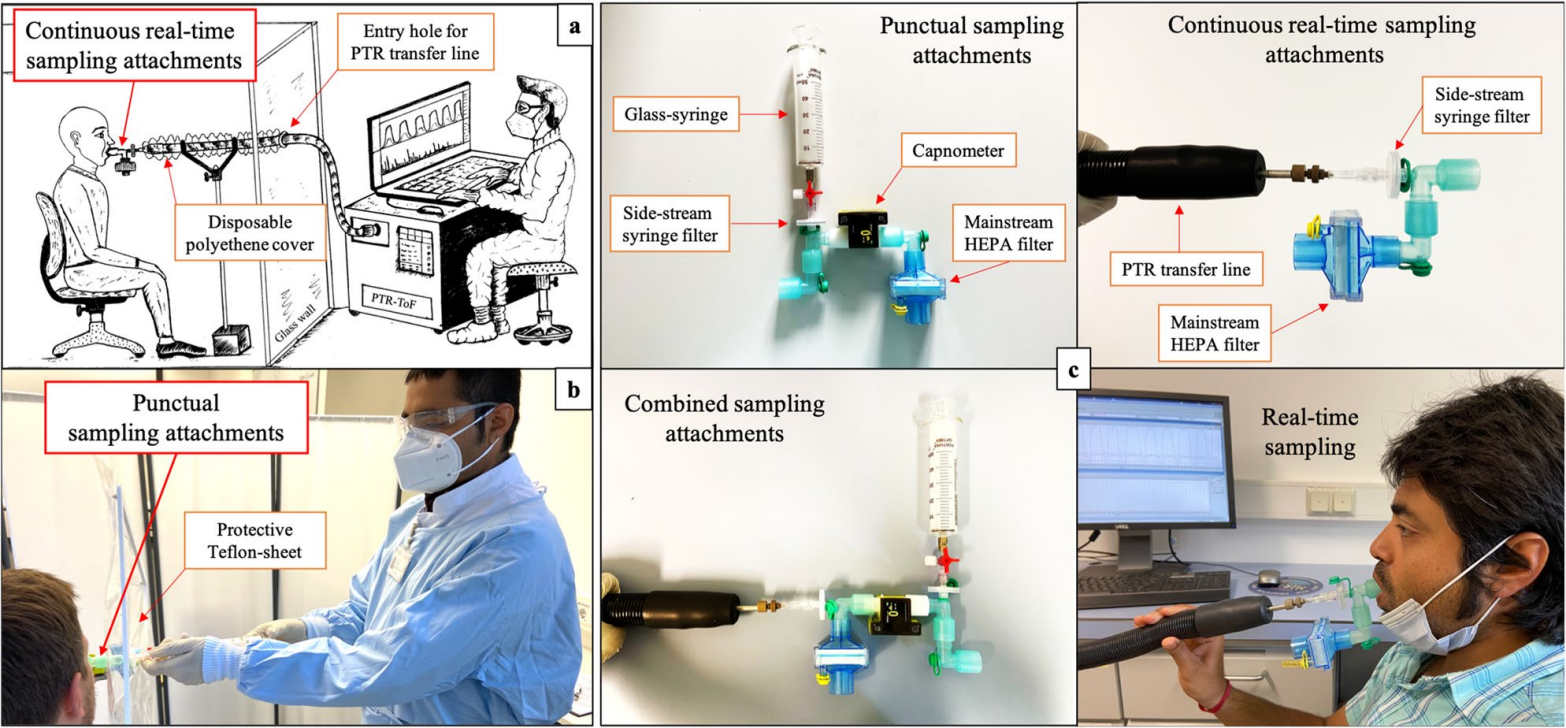
General setups for continuous real-time (**a**) and offline (**b**) sampling and customised viral filter attachments (**c**). In compliance to infection safety mandates, general setups for online and offline sampling are optimised. Punctual sampling in glass-syringe and direct continuous real-time sampling in PTR-ToF–MS are adapted via customised viral filter attachments. Combined sampling attachments to conduct simultaneous real-time and offline sampling are also presented.

The established methods and the protocol from the present study were afterwards applied in independent clinical studies e.g. on 708 subjects (aged: 39.3 ± 14.8; 355 male and 353 females; 36 COVID-19 cases, 256 healthy subjects and 416 other respiratory infections) in the actual screening scenario of the COVID-19 test center^37^ and on severely ill patients within the COVID-19 ICU of the University Medicine Rostock. Special focus was employed upon confounding effects e.g. contribution/loss VOCs by selected filter and effects of sampling flow and humidity. Effects of normal and higher respiratory rates were also tested. Operational conditions for sample storage, transport and analysis are assessed. Recommendations on general experimental setups (see Fig. 6a and b) for safe and repeatable continuous real-time and offline sampling are made. Informed consent to publish identifying information/images (in Fig. 6b and c and Supplementary Fig. S5 online) was obtained.

A PTR-ToF–MS was used for online analysis. This real-time mass-spectrometry offers direct analysis of breath volatiles, which is convenient, fast and point-of-care applicable. A series of parameters were optimized and explored for this purpose.

### Operational conditions for direct, real-time analysis with filters by PTR-ToF–MS

Breath VOCs were measured continuously via a PTR-ToF–MS 8000 (Ionicon Analytik GmbH, Innsbruck, Austria). Continuous side-stream mode of sampling via a 6 m long heated (at 75–100 °C) silico-steel transfer-line. In general, a continuous sampling flow of 20 sccm (i.e. mL/min) and a time resolution of 200 ms were applied^38^. For online measurements, the sampling flow was readjusted (to 50, 65 mL/min) to achieve breath-(phase)-resolved analysis beyond the side-stream filters. Drift tube temperature of 75 °C, voltage of 610 V and pressure of 2.3 mbar were used. The E/N ratio resulted at 139 Td. At the end of every minute, a new data file was recorded automatically and the mass scale was also recalibrated. We used the following masses for mass calibration: 21.0226 (H_3_O^+^-Isotope), 29.9980 (NO^+^) and 59.049 (C_3_H_6_O).

VOCs were measured in counts per seconds (cps) and corresponding intensities were normalised onto primary ion (H_3_O^+^) counts. Raw data were processed via PTR-MS viewer software (version 3.228). As PTR-MS continuously records both exhaled breath and inhaled room-air, the ‘breath tracker’ algorithm (based on Matlab version 7.12.0.635, R2011a) was applied to identify expiratory and inspiratory phases^32^. Here, acetone as the tracker mass was used as an endogenous substance, which has significantly higher signal intensity in expiration than in inhalation. As the mass resolution of PTR-ToF–MS (~ 4000Δm/m) can assign volatiles upon their measured protonated mass and corresponding sum formula, compound names are used while discussing results^39^. VOCs were quantified via multi-component mixture of standard reference substances^36^.

### Mainstream filters to avoid infection contamination of the clinical environment

#### Protective mainstream HEPA filter test

In all cases, we had to apply a mainstream high-efficiency particulate absorbing/HEPA filter (Ultipor BB25G Hydrophobic filter, CE 0088, PALL®, Dead space: 35 mL, Resistance of 3.5 cm H_2_O at 60 L/min) at the rare end of the breathing mouthpiece, in order to prevent any possible viral transmission (SARS-CoV-2 retention of > 99.999%) to room air from exhalation or vice versa. Nonetheless, we have also examined the real-time confounding effects from these mandatory filters.

#### Test protocol for confounding-effects

Transfer line of the PTR-ToF was connected to the sterile breathing mouthpiece in side-stream mode for continuous breath-resolved measurements of VOCs. We applied our standard sampling manoeuvre to attain steady state of breathing^2^. After two minutes of normal (spontaneous) oral breathing (by healthy volunteer in sitting position), the filter was connected to the rare end of the mainstream (i.e. after the side-stream connection of PTR transfer-line) and volunteer continued breathing through the filter for next two minutes. Similarly, room air was sampled for two minutes with and without filter for additional comparisons. Sampling attachment is presented in Fig. 6c. Test results are presented in Fig. 1a, where VOCs data are normalised onto the corresponding values from the steady state (i.e. second minute of the spontaneous respiration) of breathing without filters. The steady state of breathing is the actual comparison point for physiological and contamination effects induced by main-stream filters.

#### Mainstream sampling viral filter test

We have tested the confounding effects of a mainstream viral filter (Gibeck 0.2 μm, Filter Small S, Ref: 19512, CE 0124, Iso-Gard®, Dead space: 26 mL, Resistance of 1.6 cm H_2_O at 60 L/min, Filtration efficiency: > 99.9999% for bacteria and > 99.999% for viruses) on VOCs exhalation in order to check its applicability for breath sampling.

#### Test protocol for estimating real-time effects

Transfer line of the PTR-ToF was connected to the sterile breathing mouthpiece in side-stream mode for continuous breath-resolved measurements of VOCs. After 2 min of normal oral breathing (by healthy volunteer in sitting position) without any filter, the filter was connected to mainstream (before the side-stream connection of PTR transfer-line) and volunteer continued breathing through the filter for next two minutes. Similarly, room air was sampled for 2 min with and without filter for additional comparisons. This test was repeated with and without the rare-end HEPA filter. Sampling attachment is presented in Fig. 6c and test results are presented in Fig. 1a.

Pair-wise multiple comparisons of observed differences (in VOCs concentrations) between breathing without and with mainstream filters are tested via repeated measurements ANOVA on ranks (Dunn’s post-hoc method, *p* value ≤ 0.05). Results are presented in Supplementary Fig. S1 online.

### Side-stream filters to avoid infection contamination of the analytical equipment

The side-stream filter was short-listed via comparisons between several commercially available syringe filters. We have tested the analytical effects of small syringe filters (Sartorius PTFE 0.2 μm, Ref: 16596-HYK, Non-pyrogenic CE 1639, Ministar ®, Diameter: 26 mm, Dead space: 0.25 mL) for side-stream applications. The HYK version is with male luer-lock i.e. suitable for our additional connections. Rest of the filters were excluded due to pronounced confounding effects e.g. restriction against sampling flow, rapid accumulation of humidity and dilution of expiratory and inspiratory breath phases.

#### Test protocol for estimating analytical confounders

Transfer line of the PTR-ToF was connected to the Liquide Calibration Unit (LCU) directly and via the syringe filter for comparisons. Breath matrix adapted multicomponent VOCs standard mixture were introduced from LCU in variable concentrations and with different humidity conditions. Outputs were measured continuously and in real-time with and without filters. Loss/contribution of VOCs via filter and the effects of different inlet flows were examined. VOCs intensities obtained through measurements (via LCU and PTR-ToF–MS) without filters represent 100%, VOCs intensities obtained through measurements with syringe-filter are presented in % in relation to measurements without filter. VOCs concentration was approximately 100 ppbV and concentrations with a sampling flow rate of 20 sccm (i.e. mL/min), 50 sccm. Results are presented in Fig. 2.

#### Test protocol for continuous real-time sampling

Transfer line of the PTR-ToF was connected to the sterile breathing mouthpiece in side-stream mode for continuous breath-resolved measurements of VOCs. General setup is presented in Fig. 6a. After two minutes of normal oral breathing (by healthy volunteer in sitting position) without any filter, the syringe-filter was connected to side-stream (between mouthpiece and PTR transfer-line) and volunteer continued breathing through the filter for next two minutes. Similarly, room air was sampled for two minutes with and without filter for additional comparisons. Results are presented in Fig. 1b, where VOCs data are normalised onto the corresponding values from the steady state of breathing without filters. The steady state is the comparison point for analytical effects induced by side-stream filters.

Pair-wise multiple comparisons of observed differences (in VOCs concentrations) between breathing without and with side-stream filters are tested via repeated measurements ANOVA on ranks (Dunn’s post-hoc method, *p* value ≤ 0.05). Results are presented in Supplementary Fig. S1 online.

### Contributions or losses of VOCs via the filters, filter’s performances and stability test

Contributions and losses of VOCs via mainstream and side-stream filters are examined under sampling flows of 20, 50 and 65 mL/min. VOCs data were normalised onto the corresponding values from sampling without filters at sampling flow of 20 sccm. The steady state of breathing was compared for evaluating any contribution or loss of VOCs by the mainstream HEPA filters and side-stream syringe-filters. Results are presented in Supplementary Fig. S2 online.

Filter’s performances were examined under higher sampling flow (up to 100 mL/min) for breath phase-resolved analysis and also under higher breathing frequency (20–30 breaths/min). Results are presented in Fig. 3. Figure 3a represents side-stream sampling at inlet flows of 20, 50 and 65 mL/min via syringe-filters under normal respiratory rate of 10–14 breaths/min and Fig. 3b represents side-stream sampling at inlet flows of 65 mL/min via syringe-filters under higher respiratory rate of 20–30 breaths/min. The effects of continuous breath-resolved measurement time (i.e. 13 min of breathing with normal respiratory rate) on the stability of a syringe-filter was examined. Results are presented in Supplementary Fig. S3 online.

### Advancement of experimental setup for continuous real-time breath sampling in the screening scenario of a SARS-CoV-2 test center

Investigator/operator wore the recommended personal protective equipment (PPE) at all time. As per Fig. 6a, the area of analytical instrument and its operator were separated from the patient/subject entry and sitting area via a transparent polycarbonate flexi-glass wall (0.5 cm of thickness). Only 1 m of the PTR-transfer-line (i.e. 6 m long and heated at 75–100 °C—in order to degrade any trace-passed viral entity) entered towards patient’s side via a custom-made entry hole (circular and tightly-fitting) of the flexi-glass wall. The 1 m (i.e. at the patient side) of the transfer-line was enclosed by a sterile and disposable polyethene cover and it was changed after each participant. Subjects wore COVID-19 protective medical face masks while waiting in the test queue (by maintaining a minimum distance of 2 M from each other) and had disinfected their hands at the entrance of the SARS-CoV-2 test center. After entering the sampling area patient sat on a disinfected chair, removed his/her face masks and orally breathe in and out through the sterile and disposable mouthpiece (i.e. placed via operator before the subject/patient’s entry), which has a HEPA filter connected at the rare end. PTR-transfer line was connected to the mouthpiece in side stream mode just after our recommended side-stream syringe filter (pore size: 0.2 μm, with luer-lock). The mouthpiece (+ PTR transfer line) height was adjusted and fixed via stationary metal-clamp strands at the face height of subjects (according to his/her sitting position). Thus, participants did not touch any instrumental parts with hands and they were only allowed to encircle the sterile mouthpiece (of the customised sampling attachment) by their lips for oral breathing. As per our standard sampling manoeuvre^2^, after a minute of metronome controlled paced breathing (with respiratory rate of 12/min), volunteers continued spontaneous breathing (i.e. without the metronome) for next minutes. After sampling subjects immediately put on their face masks and disinfected their hands again at the exit of the test center. In order to avoid cross infection between subjects, used mouthpieces, viral filters and disposable polyethene cover etc. were disposed immediately after each measurement and the sampling area was disinfected and adequately ventilated before and after each participation. Our setup was successfully and safely applied for breath VOCs screening in > 700 individuals at the SARS-CoV-2 test centre of our university hospital during the surge of the delta variant^37^. Besides COVID-19 patients, we could safely profile breath VOCs from patients infected and/or co-infected by other infectious respiratory pathogens e.g. *Haemophilus influenza*, *Streptococcus pneumonia* and *Rhinovirus* etc. within our setup.

Given the fact that online analysis is difficult to implement in severely ill patients of COVID-19 or similar infectious conditions, other means of offline sampling and analysis were tested. Other detection techniques such as GC–MS also need offline samples and therefore, the relevant improvements of offline breath sampling and analysis were executed.

### Method optimization for manual offline breath sampling under COVID-19/similar conditions

#### Experiment for sampling applicability of glass-syringes

In order to check the PoC feasibility of manual CO_2_ controlled alveolar breath sampling in glass-syringes (volume 50 mL), we have connected the glass syringe behind our shortlisted filter i.e. connected at side-stream to the sampling mouthpiece. Real-time breath VOCs concentrations were measured by PTR-ToF–MS in parallel. A small portable capnograph (EMMA™ PN 3639, Ref: 605102, Masimo® Sweden AB, Danderyd, Sweden) was attached at the mainstream for visual control of the exhaled alveolar plateau (i.e. pET-CO_2_ controlled) and just after that the PTR transfer-line was also connected to the mouthpiece in side-stream mode for immediate comparison of actual breath vs. syringe sample. The end point of the mouthpiece was connected to a mainstream HEPA filter. Please refer to Fig. 6c.

#### Test measurements

After two normal exhalations (during the steady state of breathing)^2^ by the healthy volunteer, an alveolar breath (pET-CO_2_ controlled) was sampled in the glass-syringe. Thereafter, the volunteer sealed the breathing end of the mouthpiece and then within the same minute, the sampled syringe was injected back to the mouthpiece and measured as a breath by the connected PTR-ToF (see Fig. 6c). Thus, the VOCs concentrations during syringe sampling can be compared directly to the injected syringe sample. At least three syringe samples were collected from each individual.

In order to evaluate any dilution effects even from the tiny dead space of the glass syringe and syringe-filter, we have compared samples taken from same individuals, with and without flushing the instrumental dead space via 1–2 demo exhalations. Results are presented in Fig. 4.

Pair-wise multiple comparisons of observed differences (in VOCs concentrations) between direct breath vs. syringe samples are tested via repeated measurements ANOVA on ranks (Dunn’s post-hoc method, *p* value ≤ 0.05). Results are presented in Supplementary Fig. S1 online.

### Advancement of experimental setup for manual offline sampling at the COVID-19/similar test centre and in the COVID-19 intensive care unit (ICU)

As per Fig. 6b, investigator/operator wore the recommended personal protective equipment (PPE) at all time. Subjects wore COVID-19 protective medical face masks while waiting in the test queue (by maintaining a minimum distance of 2 M from each other) and had disinfected their hands at the entrance of the SARS-CoV-2 test center. Test subjects sat straight on a disinfected chair and did not touch any instrumental parts but only encircled the breathing-end of the sterile mouthpiece by his/her lips for oral breathing. In accordance to our standard sampling manoeuvre^2^, after a minute of metronome controlled paced breathing (with respiratory rate of 12/min), volunteers continued spontaneous breathing (i.e. without the metronome) for next minutes. The syringe samples were collected from the second minute onward—of the spontaneous breathing. A transparent Teflon-sheet made protective separation was used to separate the mouthpiece parts at test subject’s end from the sampling parts (i.e. Capnometer, syringe-filter, glass syringe and end-point HEPA filter) at the operator’s side. Syringes were flushed-in and -out with two exhaled breaths before taking the actual sample in order to overcome any possible dilution effects from the instrumental (syringe + filter) dead space. Immediately after sampling, the syringe (with filters attached in front) was closed via the Discofix® 3C adapter (Braun, Melsungen, Germany) adapter-system (i.e. placed between the syringe-filter and syringe) and then was detached from the syringe-filter for storage/transport. Three or more syringes were sampled from each subject. Corresponding room air was sampled separately with syringe-filter attached (via luer-lock adapter-system) for measurements and comparison of inspiratory concentrations of VOCs. After sampling subjects immediately put on their face masks and disinfected their hands again at the exit of the test center. Used mouthpieces, viral filters and disposable polyethene cover etc. were disposed immediately after each measurement and the sampling area was disinfected and adequately ventilated before and after each participation.

### Analysis protocol for syringe samples via PTR-ToF–MS and Needle trap microextraction (NTME) coupled with GC–MS

#### Injection of syringe sample with a uniform manual push over a min into PTR transfer-line

Our preoptimized instrumental parameters for continuous VOCs analysis are described earlier. Sampled syringes were analysed via direct injection to PTR transfer line. The constant transfer-line flow of 20 mL/min was not enough to create adequate vacuum within the syringe to sample those uniformly. Therefore, we pushed the plunger to inject 50 mL volume over one minute manually. In order to do so, an automated volume/time-controlled injector device can be used. This will overcome the problem of unavoidable vaping/condensation (storage/transport) driven mechanical resistance at the contact surface of the barrel and plunger of a glass syringe. PTR data of syringe samples was analysed as an average over one minute.

#### NTME of VOCs from collected syringe samples via automated flow-volume controlled sampling box

Needle trap devices (NTDs) were connected to an automatic alveolar sampling box (PAS Technology Deutschland GmbH, Magdala, Germany). An IN-stopper coupled with Discofix® 3C adapter (Braun, Melsungen, Germany) was connected and NTDs were pierced through the IN-stopper membrane into the 50 mL glass syringes. 20 mL of samples (breath gas and room air) was sampled unidirectionally.

### Comparisons of data for inter-syringe variations, effects of storage conditions on VOCs stability

#### Transport of sampled syringes from bedside to bench

In order to prevent temperature change or condensation effects we have stored glass-syringes in tightly-sealed Styrofoam boxes. Pre-heated gel-bags were kept inside these boxes to maintain the temperature at around 37 °C. All empty/unused and sampled syringes were kept in the same temperature during storage and transport. Syringe samples were also tested by keeping in an incubator to maintain constant temperature at around 37 °C. Results are presented in Fig. 5, where VOCs data are normalised onto the corresponding values from the syringe sample i.e. analysed immediately after sampling.

#### Effects of sample storage time (15–120 min)

In order to evaluate the effects of sample storage time, we have sampled multiple syringes from same individuals and stored them for 15, 30, 60 and 120 min respectively prior to analysis. Any loss, degradation, clustering or condensation of VOCs were analysed via online analysis. Results are presented in Fig. 5, where VOCs data are normalised onto the corresponding values from the syringe sample i.e. analysed immediately after sampling. Pair-wise multiple comparisons of observed differences (in VOCs concentrations) between instantly analysed syringe sample vs. syringe samples stored for different time and under different conditions are tested via repeated measurements ANOVA on ranks (Dunn’s post-hoc method, *p* value ≤ 0.05). Results are presented in Supplementary Fig. S1 online.

#### Inter-sample variations and repeatability

Differences and variations in VOCs concentrations between samples (at the steady state of breathing) from same individuals were examined in order to check the robustness and repeatability of our above-described method. Statistically significant differences were tested via repeated measurements ANOVA on ranks (Dunn’s post-hoc method, *p* value ≤ 0.05). Results are presented in Supplementary Table S1 online.

## Supplementary Information

Supplementary Information.

## Data Availability

All data generated or analysed during this study are included in this published article (and its Supplementary Information files).
