# Supplementary material for: Advanced setup for safe breath sampling and patient monitoring under highly infectious conditions in the clinical environment
Source: Sci Rep. 2022 Oct 26;12:17926. doi: 10.1038/s41598-022-22581-7 (PMC9606119; doi:10.1038/s41598-022-22581-7)
Supplement: Supplementary file 1 — Supplementary Information. [file 41598_2022_22581_MOESM1_ESM.pdf]

Advanced setup for safe breath sampling and patient monitoring under highly infectious conditions in the clinical environment

Pritam Sukul[1]*, Phillip Trefz[1], Jochen K Schubert[1], Wolfram Miekisch[1]

[1]*Rostock Medical Breath Research Analytics and Technologies (ROMBAT), Dept. of Anaesthesiology and Intensive Care, University Medicine Rostock, Schillingallee 35, 18057 Rostock, Germany*

*Corresponding authors: pritam.sukul@uni-rostock.de

Supplementary Information

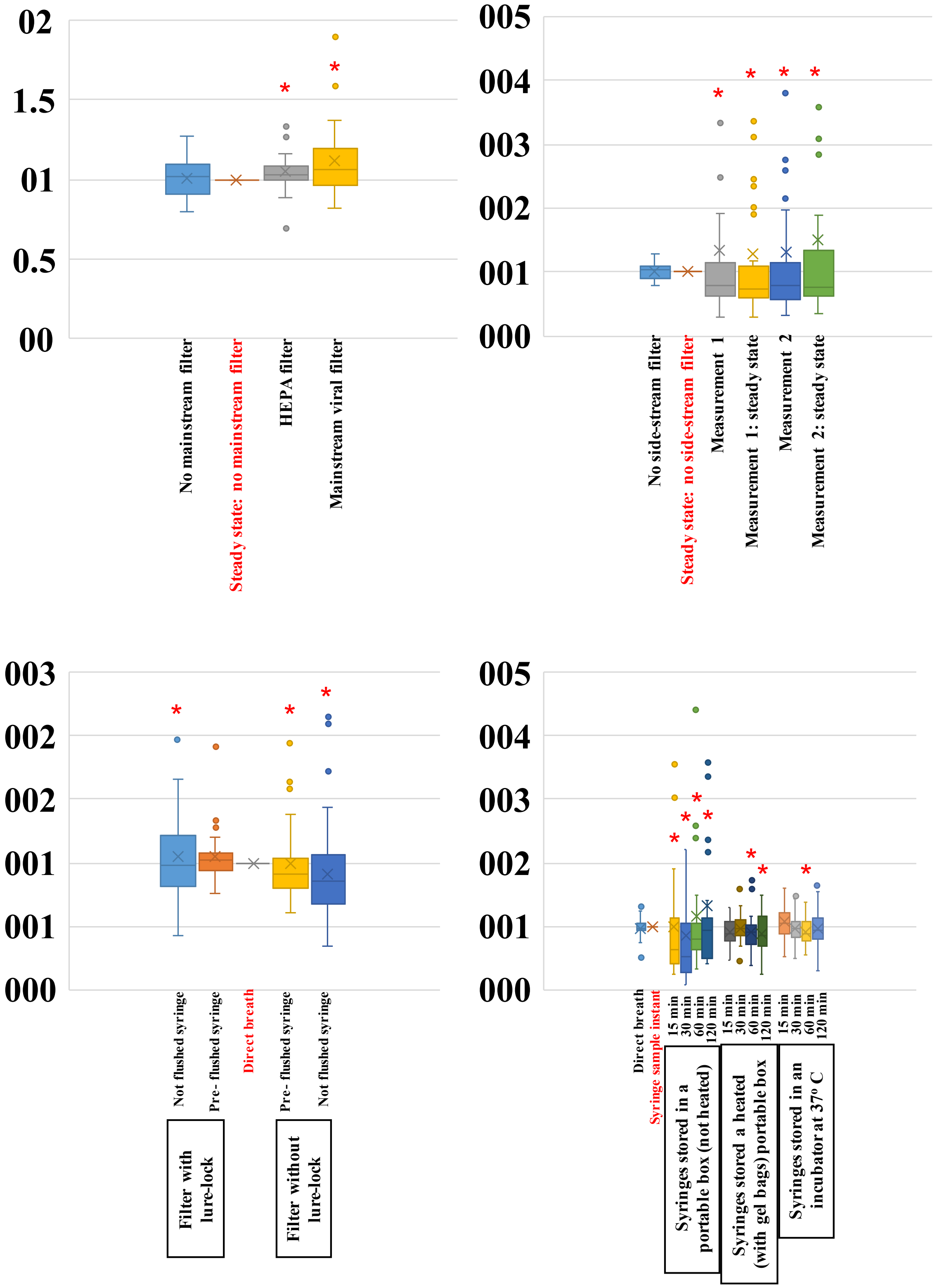

Supplementary figure S1: Statistical significances of observed differences in Figure 1a, 1b, 4 and 5 are presented. Statistical significance are tested via repeated-measurements ANOVA on ranks (Dunn's post-hoc method, p value ≤ 0.05). Substances produced by the filters are excluded from statistical analysis. Within each plot, comparison point is marked via red coloured legend at the X-axis and significant differences with respect to that comparison point are presented via red coloured '*'.

						Experiment 1						Experiment 2					
Protonated/Charged VOCs	Mass	M1 - No filter_20sccm	M2 - No filter_20sccm_SS	M1 - HEPA_20sccm	M2 - HEPA_20sccm	M1 - HEPA+SF_20sccm	M2 - HEPA+SF_20sccm	M1 - HEPA+SF_50sccm	M2 - HEPA+SF_50sccm	M1 - HEPA+SF_65sccm	M2 - HEPA+SF_65sccm	M1 - HEPA+SF_20sccm	M2 - HEPA+SF_20sccm	M1 - HEPA+SF_50sccm	M2 - HEPA+SF_50sccm	M1 - HEPA+SF_65sccm	M2 - HEPA+SF_65sccm
cluster_h2oH+	37.02755	0.9	1.0	0.7	0.5	2.6	2.4	1.3	1.9	1.8	2.0	2.1	2.8	1.9	1.9	1.9	2.0
(C2H5O)+	45.03349	1.1	1.0	1.0	1.0	4.8	4.8	1.7	1.5	1.3	1.2	1.8	2.8	4.9	3.2	2.0	1.6
(C2H6NH)H+	46.064	0.8	1.0	1.0	1.1	2.7	2.4	1.1	1.1	0.9	0.7	1.0	1.7	2.5	1.9	1.1	1.1
(C2H5NH2)H+	46.065	0.8	1.0	1.0	1.0	3.3	2.8	1.1	1.1	1.1	0.8	1.2	2.0	2.5	2.0	1.3	1.2
(H3O3P)H+	82.98927	2.0	1.0	1.9	1.8	10.9	10.1	6.5	6.0	4.7	3.7	12.2	10.5	8.7	6.5	6.1	4.6
(C2HF3)H+	83.01031	1.4	1.0	1.1	1.1	8.4	8.3	4.7	4.2	3.8	3.5	9.9	9.5	6.8	5.3	4.0	4.0
(C6H10)H+	83.08553	1.1	1.0	1.1	0.8	3.3	3.4	3.4	3.0	2.7	2.7	3.8	3.6	3.6	3.5	3.5	3.2
(C4H6O2)H+	87.04406	1.1	1.0	1.1	1.0	10.0	9.3	5.2	4.0	2.9	2.5	10.2	15.0	14.2	8.1	5.3	4.2
(C5H10O)H+	87.08044	0.9	1.0	1.1	1.1	4.4	4.9	2.1	2.2	1.7	1.5	4.1	6.6	6.5	4.0	2.6	2.2
(C5H12O)H+	89.09609	0.4	1.0	1.1	1.2	0.5	0.6	0.3	0.4	0.4	0.3	0.3	0.3	0.3	0.2	0.2	0.2
(C7H12)H+	97.10118	0.9	1.0	1.1	0.9	4.4	4.3	4.6	4.2	4.0	3.7	4.4	4.6	5.3	5.1	5.1	4.8
(C6H12O)H+	101.09608	0.7	1.0	0.9	0.9	2.2	2.5	1.2	1.6	1.4	1.2	2.2	2.8	2.3	2.0	1.5	1.4
(C6H14O)H+	103.11174	0.5	1.0	1.3	1.1	0.5	0.6	0.4	0.5	0.3	0.3	0.6	0.8	0.9	0.6	0.5	0.3
(C5H14N2)H+	103.12298	0.4	1.0	1.2	1.0	0.5	0.6	0.4	0.5	0.4	0.4	0.5	1.0	0.9	0.8	0.6	0.2
(C4H8OS)H+	105.03687	0.8	1.0	1.0	1.0	16.4	13.4	10.6	6.3	4.8	3.9	15.5	21.4	22.9	14.1	10.1	7.8
(C7H7N)+	105.0573	0.7	1.0	1.0	1.0	28.8	25.3	14.7	11.8	8.4	6.8	26.3	38.9	42.0	24.5	17.4	13.3
(C8H8)H+	105.06987	0.8	1.0	1.0	1.1	35.5	32.2	16.6	15.1	10.9	8.7	32.0	48.0	50.9	30.0	21.3	16.0
(C8H10)+	106.07771	0.7	1.0	1.0	0.8	8.3	7.5	3.8	4.3	3.4	3.7	7.7	12.1	10.5	6.4	5.0	4.4
(C7H6O)H+	107.04913	1.1	1.0	1.6	1.0	6.5	6.1	3.9	3.3	2.9	2.7	6.0	6.2	4.9	4.2	3.9	3.5
(C7H13N)+	111.10425	1.0	1.0	2.4	1.6	3.3	3.2	3.0	2.7	2.6	2.5	3.1	3.2	3.5	3.4	3.4	3.1
(C8H14)H+	111.11681	1.0	1.0	2.0	1.4	4.3	4.3	3.8	3.8	3.7	3.3	4.1	4.4	4.9	4.8	4.7	4.5
(C7H13N)H+	112.11207	0.6	1.0	2.0	1.2	2.2	2.3	2.1	2.3	1.8	2.1	2.7	2.6	2.6	2.7	2.7	2.4
(C7H6N2)H+	119.06039	1.2	1.0	1.0	1.3	7.7	5.9	6.4	4.6	3.3	3.5	7.3	10.6	12.3	7.9	6.5	5.1
(C9H10)H+	119.08553	1.3	1.0	1.3	1.2	11.3	9.9	8.2	7.8	5.9	6.7	11.3	16.7	18.7	12.6	10.2	8.9
(C8H9N)H+	120.08076	0.6	1.0	0.9	0.6	3.8	3.3	3.6	2.4	2.9	2.1	3.3	5.1	5.5	4.2	3.6	3.6
(C10H12)H+	133.10118	1.1	1.0	1.6	1.0	3.8	3.6	2.8	3.3	2.2	2.3	3.5	5.1	6.0	4.2	3.6	2.9
(F6S)H+	146.96976	0.3	1.0	0.6	0.7	1.4	0.7	1.3	0.4	1.0	0.7	1.3	0.8	1.1	1.4	1.0	0.9
(C10H12O)+	148.08827	0.5	1.0	0.4	0.9	1.1	0.9	1.6	1.6	1.0	1.1	1.2	0.8	0.9	0.7	0.8	0.7
(C11H16)+	148.12465	0.4	1.0	0.8	0.8	1.6	1.3	1.6	1.4	1.3	1.0	1.5	1.5	2.1	1.3	1.5	1.9
(C12H10)H+	155.08554	1.3	1.0	6.8	5.3	2.1	1.9	1.7	1.5	1.5	1.5	1.1	1.2	1.2	1.1	1.1	0.9
(C9H14O2)H+	155.10666	1.0	1.0	5.3	4.2	1.8	1.7	1.4	1.3	1.3	1.3	1.1	1.2	1.2	1.0	1.0	0.9
(C11H17N)+	163.13556	0.8	1.0	1.2	1.6	3.4	3.1	3.4	3.4	2.5	2.6	3.1	3.2	4.0	3.8	3.5	3.5
(C12H18)H+	163.14813	0.8	1.0	1.4	1.6	4.1	3.7	3.8	3.7	3.1	3.0	3.4	4.0	4.7	4.7	4.3	4.0
(C10H12O2)H+	165.091	1.0	1.0	2.0	2.5	3.7	2.1	1.9	1.5	1.5	1.2	2.0	2.1	2.3	1.4	1.7	1.4
(C13H18)H+	175.14813	0.6	1.0	1.0	0.9	4.8	6.4	4.4	3.6	3.2	3.0	7.5	6.3	7.7	6.8	5.3	4.4
(C11H15NO)H+	178.12262	0.7	1.0	2.3	2.1	2.6	1.8	2.1	1.0	1.7	1.8	1.3	1.6	1.6	2.6	1.1	1.2
(C12H20O)+	180.15086	0.5	1.0	1.9	1.1	1.9	1.3	3.2	1.5	1.2	2.3	2.2	2.3	1.9	1.4	2.7	1.3
(C14H18)H+	187.14813	0.6	1.0	1.0	1.4	4.7	4.3	3.5	4.1	3.7	3.5	3.9	4.8	5.3	5.4	4.7	4.9
(C14H20)H+	189.16377	0.5	1.0	1.1	0.9	13.0	12.4	12.1	10.3	9.4	9.1	13.5	13.0	16.4	15.4	15.0	13.1
(C13H18O)H+	191.14304	0.8	1.0	0.9	1.2	2.6	1.9	2.9	2.3	1.7	1.6	3.3	3.0	3.2	1.9	2.6	2.2
(C15H12)H+	193.10118	0.7	1.0	1.0	0.9	2.2	1.7	2.0	1.6	1.4	0.8	2.0	1.6	1.6	2.0	1.8	1.0
(C13H16N2)H+	201.13863	1.0	1.0	1.3	0.9	5.4	4.5	6.2	4.6	4.6	4.3	4.7	5.0	6.6	6.6	6.0	6.7
(C12H28N2)H+	201.23253	1.2	1.0	1.7	1.4	12.3	14.3	8.7	13.5	13.4	9.8	10.2	10.8	14.1	17.4	17.1	12.6
(C16H10)H+	203.08554	1.0	1.0	1.0	2.2	3.2	2.3	3.8	2.6	1.8	3.0	2.3	3.6	4.0	5.1	2.4	4.7
(C13H18N2)H+	203.15427	1.0	1.0	1.3	1.7	13.8	10.5	15.8	10.7	9.6	11.3	12.7	13.4	17.5	16.1	16.4	17.2
(C15H24)+	204.18723	0.7	1.0	1.7	1.5	5.9	5.8	5.1	5.6	4.2	5.9	5.6	7.7	7.8	7.0	7.6	6.5
(C12H19N3)+	205.15735	1.3	1.0	1.0	1.3	5.2	4.6	6.5	4.7	4.4	4.3	6.1	5.4	6.6	6.9	6.8	5.1
(C15H24)H+	205.19507	1.0	1.0	1.3	1.3	5.5	5.5	5.0	4.9	4.5	4.7	6.2	6.1	7.0	7.1	7.1	5.8

Supplementary figure S2: Contributions/losses of VOCs via mainstream and side-stream filter under different sampling flows. Y-axis represents protonated VOCs masses of interest. X-axis represents experimental conditions. VOCs data were normalised onto the corresponding values from sampling without filters. The second minute of steady state (M2 – No filter_20sccm_SS) at sampling flow of 20 sscm is the actual comparison points for evaluating any contribution or loss of VOCs by the mainstream (HEPA) filters and side-stream syringe-filters (SF). Changes in colour represents relative differences in concentrations. Red and blue colour represents relatively high and low values, respectively. Confounding effects under the sampling flows of 20, 50 and 65 sccm were tested. Repeated experiments demonstrate systematic effects.

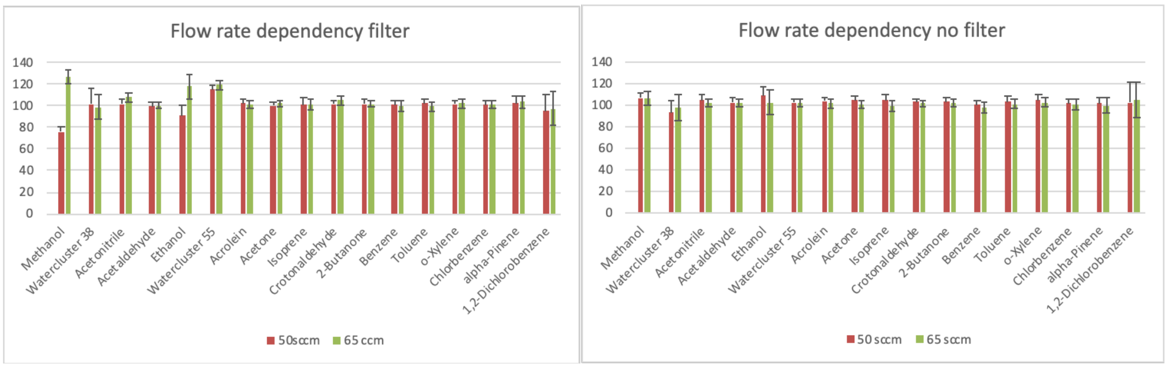

Supplementary figure S3: Comparison between flow-dependency of VOCs at inlet flows of 50 sccm and 65 sccm. VOCs intensities obtained through measurements with and without syringe filter are presented. VOCs concentration was approximately 100 ppbV. Red bars represent measurements with a sampling flow rate of 50 sccm (i.e. mL/min), Green bars represent measurements with a sampling flow rate of 65 sccm.

Run-time of syringe filters: every second minute of breath-resolved profiles of acetone depicting the dilution of inspiratory and expiratory phases after 5th minute of continuous measurements

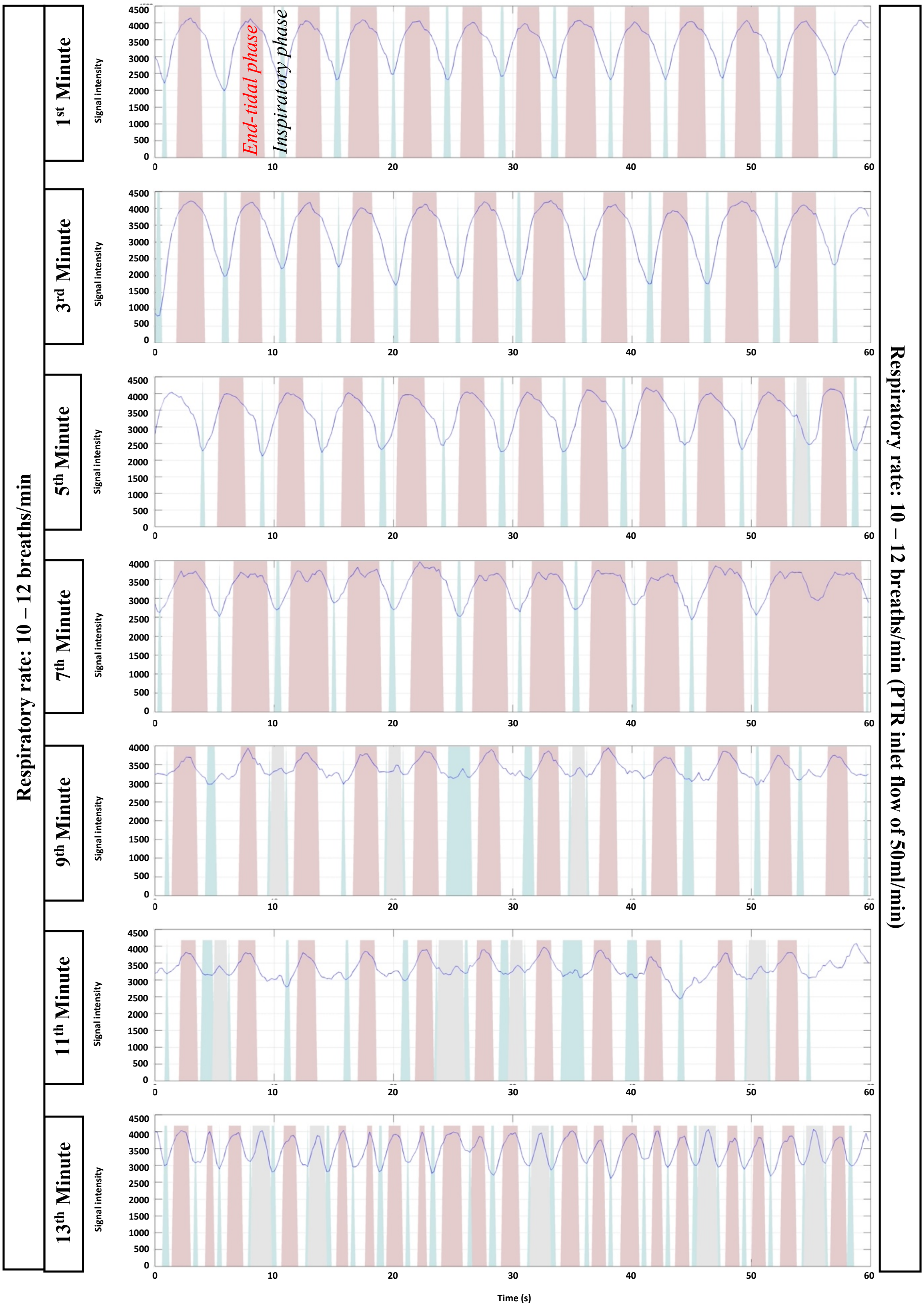

Supplementary figure S4: Effects of continuous breath-resolved measurement time on the stability of a syringe filter. Every 2nd minute of breath tracker profile from 13 min of continuous sampling under normal respiratory rate (10 -12 breaths/min) are presented. Breath acetone profiles (blue waveform) indicates that the distinct separations between inspiratory and expiratory breath phases starts to dilute after the 5th minute of sampling.

Scheme of advanced setup for safe breath sampling and analysis

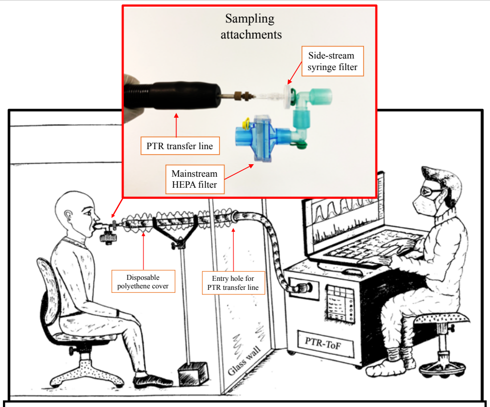

Continuous real-time sampling setup

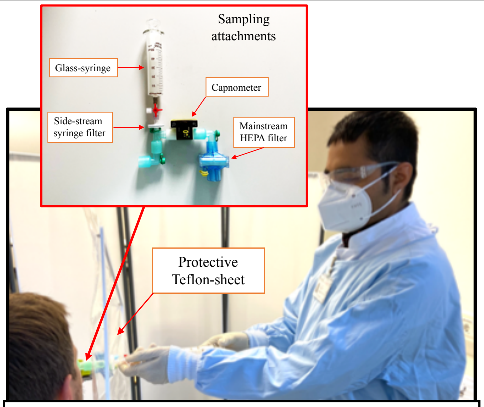

Punctual offline sampling setup

Breath sampling protocol

Posture: sitting position (if not severely ill or mechanically ventilated patient)
Breathing manoeuvre: 1 min of paced breathing (respiratory rate: 10 – 12 /min) followed by 3 – 4 min of spontaneous breathing

Direct sampling parameters

PTR sampling flow: 65 – 100 mL/min
PTR transfer line temperature: 75 – 100 °C
PTR time resolution: 200 ms
PTR drift tube temperature: 75 °C
PTR drift tube voltage: 610 V
PTR drift tube pressure: 2.3 mbar
PTR E/N ratio: 139 Td

Syringe sampling parameters

Syringe: 50 – 100 mL glass syringe
Alveolar sampling: pET-CO_2 controlled
Sampling at: 2nd min onward of the spontaneous breathing
Flush syringe: by 1 – 2 alveolar breaths
Alveolar breath samples: ≥ 3 syringes
Room air sample: ≥ 3 syringes

Mandatory safety attributes

Subjects: do not touch instrument (only mouthpiece by lips), use face-mask before & after sampling, disinfect hands at the entry and exit of the test center
Investigators: use PPE, dispose all single-use materials, disinfect subject's chair and ventilate sampling area

Filter performance & stability

Respiratory rate: up to 20 breaths /min
Continuous measurement: up to 5 min
Recommended inlet flow: 100 mL/min
VOCs Contribution/loss: substance-specific
Variability in repeated measures: < 10%

- Continuous breath-resolved assignments of VOCs to inspiratory and expiratory phases via '*Breath Tracker*' algorithm

Sample storage & transport

Storage: heated Styrofoam box/incubator
Storage temperature: 37 °C
Storage time: up to 1 hour
Incubation at 37 °C: 15 min before analysis

- Direct injection of 50 mL to PTR-ToF-MS manually or via injector over 1 min
- 20 mL sampled unidirectionally to NTME for GC-MS analysis

Supplementary figure S5: Schematic overview of advanced setup for safe breath sampling and analysis. Process of online and offline breath sampling and analysis are presented along with corresponding analytical parameters and associated infection safety attributes. Informed consent to publish identifying information/image was obtained.

VOCs	Mass	Mean of differences (%)	SDs (±)
**(CH4O)H+**	**33.03**	5.09	2.91
**(H2S)H+**	**35.00**	7.42	3.15
**cluster_h2oH+**	**37.03**	3.24	1.17
**(C2H3N)H+**	**42.03**	5.11	1.10
**(CH2O2)H+**	**47.01**	2.26	0.99
**(C2H6O)H+**	**47.05**	7.41	1.53
**(CH4S)H+**	**49.01**	5.07	1.11
**(C3H4O)H+**	**57.03**	1.33	1.70
**(C4H8)H+**	**57.07**	1.29	1.22
**(C3H6O)H+**	**59.05**	4.57	1.32
**(C4H10)H+**	**59.09**	9.37	3.79
**(C2H4O2)H+**	**61.03**	5.03	3.95
**(C3H8O)H+**	**61.06**	4.65	1.12
**(C2H6S)H+**	**63.03**	9.71	5.60
**(C4H4O)H+**	**69.03**	1.76	0.24
**(C5H8)H+**	**69.07**	10.25	3.16
**(C4H6O)H+**	**71.05**	2.97	2.08
**(C5H10)H+**	**71.09**	2.82	2.64
**(C4H8O)H+**	**73.06**	2.37	2.92
**(C3H6O2)H+**	**75.04**	6.44	6.36
**(C3H8O2)H+**	**77.06**	5.51	5.55
**(C6H6)H+**	**79.05**	4.84	4.73
**(C6H8)H+**	**81.07**	4.40	2.28
**(C6H10)H+**	**83.09**	1.73	0.76
**(C4H6O2)H+**	**87.04**	5.11	2.96
**(C3H4O3)H+**	**89.02**	10.30	2.33
**(C4H8S)H+**	**89.04**	7.48	1.81
**(C4H8O2)H+**	**89.06**	5.18	1.35
**(C4H10S)H+**	**91.06**	5.34	4.84
**(C7H8)H+**	**93.07**	5.03	3.93
**(C6H6O)H+**	**95.05**	4.54	5.04
**(C5H8O2)H+**	**101.06**	6.26	0.21
**(C10H16)H+**	**137.13**	10.79	7.03

Supplementary Table S1: Comparisons between absolute abundances of exhaled VOCs measured repeatedly by customised viral filter attachments. In order to determine the repeatability, VOCs were measured at the steady state of breathing in same individuals repeatedly via our customised viral filter attachments (i.e. mainstream HEPA filter and side-stream syringe filter as shown in Figure 1). Mean (from individuals) values of observed differences (%) in absolute abundances of VOCs between measurements and corresponding SDs are presented. Overall differences in VOCs concentrations remained statistically insignificant in repeated-measurements ANOVA on ranks (Dunn's post-hoc method, p value ≤ 0.05) test.
